# Rift Valley Fever Outbreak with East-Central African Virus Lineage in Mauritania, 2003

**DOI:** 10.3201/eid1307.061487

**Published:** 2007-07

**Authors:** Ousmane Faye, Mawlouth Diallo, Djibril Diop, O. Elmamy Bezeid, Hampathé Bâ, Mbayame Niang, Ibrahima Dia, Sid Ahmed Ould Mohamed, Kader Ndiaye, Diawo Diallo, Peinda Ogo Ly, Boubacar Diallo, Pierre Nabeth, François Simon, Baïdy Lô, Ousmane Madiagne Diop

**Affiliations:** * Institut Pasteur de Dakar, Dakar, Sénégal; † Institut National de Recherche en Santé Publique, Nouakchott, Mauritania; ‡ Centre National d’Elevage et de Recherche Vétérinaire, Nouakchott, Mauritania

**Keywords:** Rift Valley fever, outbreak, virus lineage, emergence, field investigations, human, animals, mosquitoes, research

## Abstract

In October 2003, 9 human cases of hemorrhagic fever were reported in 3 provinces of Mauritania, West Africa. Test results showed acute Rift Valley fever virus (RVFV) infection, and a field investigation found recent circulation of RVFV with a prevalence rate of 25.5% (25/98) and 4 deaths among the 25 laboratory-confirmed case-patients. Immunoglobulin M against RVFV was found in 46% (25/54) of domestic animals. RVFV was also isolated from the mosquito species *Culex poicilipes*. Genetic comparison of virion segments indicated little variation among the strains isolated. However, phylogenetic studies clearly demonstrated that these strains belonged to the East-Central African lineage for all segments. To our knowledge, this is the first time viruses of this lineage have been observed in an outbreak in West Africa. Whether these strains were introduced or are endemic in West Africa remains to be determined.

## Abstract 2

Phylogenetic studies demonstrated that outbreak strains belonged to the East-Central African lineage.

Rift Valley fever (RVF) is an acute febrile viral disease that affects small domestic ruminants (*1*) and humans. The disease in animals is characterized by high rates of abortion and death of young ruminants (*2*). In humans, the symptoms are usually mild, but in severe cases, hemorrhages, meningoencephalitis, retinopathy, and sometimes death can occur (*3*). The disease is widespread in Africa, mainly in the sub-Saharan region but also in Egypt. In 2000, outbreaks were recorded for the first time outside of the African continent, in Saudi Arabia and Yemen (*4*). RVF virus (RVFV) belongs to the family *Bunyaviridae*, genus *Phlebovirus* genus, and its genome consists of 3 negative single-stranded RNA segments referred to as L (large), M (medium), and S (small) (*5*).

In West Africa, the first extensive RVF outbreak recorded to date occurred in Mauritania in 1987 and resulted in 220 human deaths (*6*). After this outbreak, an active surveillance system led to the detection of several animal cases in Mauritania, Senegal, and other West African countries (*7*–*9*). Furthermore, during interepizootic periods, RVFV has been repeatedly isolated from different mosquito species in Senegal, Burkina Faso, and Nigeria (*10*–*12*,). During 1998, an outbreak of RVF occurred in southeastern Mauritania, resulting in 300 to 400 human cases and 6 deaths (*13*).

In Mauritania in 2000, health authorities established a National Disease Surveillance System (NDSS) by using sentinel herds in 5 geographic regions and a notification system of hemorrhagic fever in medical healthcare centers. This NDSS was implemented in collaboration with the Centre National d’Hygiène, the “Centre National d’Elevage et de Recherche Vétérinaire” in Mauritania, and the Institut Pasteur de Dakar in Senegal. The value of the NDSS was further reinforced after an outbreak of Crimean-Congo hemorrhagic fever in Mauritania in February 2003 (*14*) for which RVFV was identified in animal and human serum specimens collected in September and October 2003. We describe the results of a multidisciplinary investigation to determine extent of the outbreak and the key factors responsible for RVFV reemergence in Mauritania**.**

## Materials and Methods

### Case Definitions

A suspected human RVFV case-patient was defined as a person with fever associated or not with hemorrhagic, jaundice, or neurologic symptoms or any person who died who had had overt hemorrhagic fever symptoms from September through December 2003. A confirmed human RVFV case-patient was defined as a person for whom laboratory tests confirmed an acute or recent RVFV infection, e.g., by >1 of these results: immunoglobulin M (IgM), reverse transcription–PCR (RT-PCR) or virus isolation positive results. A human contact was defined as any person or relative who had been directly in contact with a confirmed human or animal case-patient, or with a person who died who had had overt hemorrhagic fever symptoms from September through December 2003.

### Field Investigations

#### Study Sites

**Figure 1:**
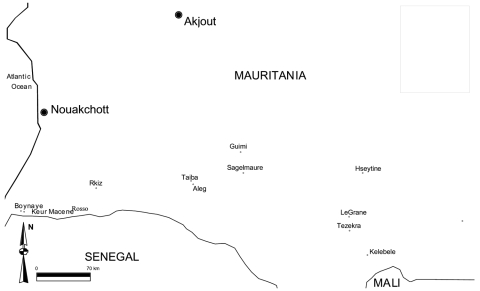
Locations of the study sites.

Nine localities belonging to 3 administrative provinces were visited (Figure 1): Keur Macène and Rkiz (Trarza Province), Makhtar Lahjar, Guimi, Taïba, and Sagle Moure (Brakna Province), Legrane, Kélébélé, and Hseytine (Assaba Province). These localities were chosen because they had confirmed human or animal cases.

#### Human Investigations

In affected areas, the investigation was conducted under the supervision of the chief of the sanitary district. For each case, venous or capillary blood samples were collected into dry tubes or onto filter papers, respectively. A thick blood smear was also taken from all suspected case-patients for differential diagnosis of malaria. An interview in which information was gathered about sex, age, date of fever onset, and hemorrhagic signs was conducted for all case-patients and their contacts.

#### Animal Investigations

All domestic animals living in the close vicinity of suspected or confirmed case-patients were included in the study. Every blood sample was accompanied by an investigation form specifying the species, age, and localization of the animal during the month before the investigation and, for female animals, a history of pregnancies.

#### Entomologic Investigations

Adult mosquitoes were collected in CDC light-traps (*15*), with or without CO_2_, which were placed close to water points or in sheepfolds, respectively; animal-baited traps were placed in the houses of persons with suspected or confirmed cases. Mosquitoes were frozen and subsequently identified on a chilled table by using morphologic keys (*16*,*17*). They were classified into monospecific pools, stored in liquid nitrogen, and transported to the laboratory, where they were kept at –80°C until virus isolation was attempted.

### Laboratory Tests

#### Serologic Studies

All human and animal samples were tested for evidence of IgG and IgM by using an ELISA technique (*18*,*19*). Serum specimens were considered positive for antibodies if the difference between the sample and control optical densities was >3 standard deviations above the mean of the negative controls.

#### Molecular Studies

Viral RNA was extracted from serum of suspected case-patients by using the QIAamp RNA kit (QIAGEN, Inc. Chatsworth, CA, USA) and RT-PCR was done by using the Titan One-Step RT-PCR System (Roche Diagnostics, Mannheim, Germany), according to the recommendations of the manufacturers. The primers NS3a (nt 710–729; 5′-ATGCTGGGAAGTGATGAGCG-3′) and NS2g (nt 61–80; 5′-TGATTTGCAGAGTGGTCGTC-3′) were used to amplify a 669-nt region of the virus S segment region encoding the NSs protein. The primers MRV1a (nt 772–790; 5′-CAAATGACTACCAGTCAGC-3′) and MRV2g (nt 1563–1580; 5′-GGTGGAAGGACTCTGCGA-3′) were used to amplify a 809-nt region of the virus M segment region encoding the G2 protein. Primers Wag (nt 4440–4457; 5′-ATTCTTATTCCCGAATAT-3′) and Xg (nt 4634–4651; 5′-TTGTTTTGCCTATCCTAC-3′) were used to amplify a 212-nt region of the L segment (*20*–*22*). The PCR products were purified on agarose gel and directly sequenced by using the Sanger method with an ABI 377 sequencer (Applied Biosystems, Foster City, CA, USA). Phylogenetic trees on the partial sequences of the S (601 nt), M (726 nt), and L (121 nt) RNA segments were constructed by using the maximum likelihood method (PAUP* 4.0, Sinauer Associates Inc., Sunderland, MA, USA).

#### Virologic Studies

Virus isolation was performed at the World Health Organization (WHO) Collaborating Center for Arboviruses (www.pasteur.fr/recherche/banques/crora), Institut Pasteur de Dakar on mosquitoes and serum collected from humans by inoculating the virus into suckling mice and a mosquito cell line (AP61). Virus identification was performed by an indirect immunofluorescence assay that used polyclonal and monoclonal antibodies. The identification of virus isolates was confirmed by complement fixation (*23*).

#### Parasitologic Test

To rule out malaria infection, thick blood smears from patients with suspected cases were Giemsa-stained. The ratio of parasite (*Plasmodium falciparum*) to leukocytes was estimated in 200 fields based on a mean leukocyte count of 8,000/µL of blood.

## Results

### Human Cases

**Figure 2:**
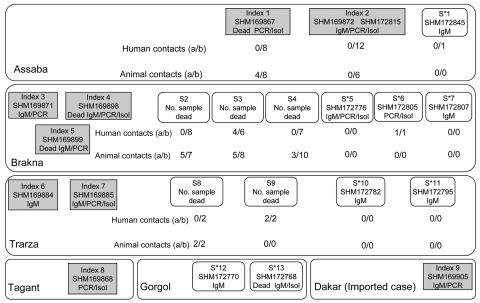
Investigation of human and animal contact around index and suspected case-patients (S) from Mauritania in 2003. For each case-patient (represented as a box), PCR, immunoglobulin M (IgM), or isolation (Isol)-positive test results are indicated below the sample number (e.g., 169867).(a/b), no. IgM positive/no. tested; S*, suspected case-patient before field investigation and subsequently confirmed positive by laboratory tests.

The different cases recorded and the linkages between them are represented in Figure 2. The 9 confirmed case-patients, identified before the investigation, were from the Assaba, Brakna, Trarza, and Tagant provinces. Subsequent discussions with the head of the sanitary district enabled the localization of the residences and the relatives for 2 of them (index case-patients 1 and 2). Several confirmed cases-patients (index case-patients 3 to 9) and their relatives were not found due to the great distances between localities or nomadic behaviors of populations. However, for all suspected case-patients (S), further investigation was conducted in the provinces where the confirmed case-patients lived.

In total, 98 persons (66 contacts, 23 with suspected cases, and 9 with confirmed cases) were included in this study. Of these persons, 25.5% (25/98) had evidence of recent RVFV infection (i.e., presence of IgM, viral RNA or virus, or >1 of these results), and 10% had evidence of past infection (i.e., presence of IgG alone). Seven viral strains were isolated. For the 25 patients who were recently infected (9 with confirmed cases before the investigation, 6 contacts, and 10 with suspected cases at the time of investigation), the median age was 21 years (range 7–50 years), 4 died, and 16 had hemorrhagic signs (hematemesis, vaginal bleeding, severe hemoptysis, bleeding from the gums and venipuncture sites, petechial rashes, and ecchymoses of the skin). Among the 23 suspected case-patients from whom blood samples were collected, 10 were infected by RVFV; only 2 patients were positive for malaria parasites.

In Assaba Province, 2 confirmed case-patients were recorded before the investigation (index case-patients 1 and 2), and 1 suspected case-patient (S1) was found during the investigation. The index case-patient 1 was dead at the time of the investigation; however, RVFV (strain SHM169867) was isolated from a blood sample taken during his illness. No RVFV infection was noticed in contact persons associated with this patient. However, IgM against RVFV was detected in animals living in and near the residence of index case-patient 1. For index case-patient 2 (infected with the strain SHM169872), a second blood sample was taken, and presence of IgM antibodies against RVFV was confirmed. No evidence of RVFV infection was detected in contact persons or in animals living in or near the residence of this patient. The suspected case-patient 1 was identified in Kiffa Hospital, where he was admitted on November 3, 2003. This 50-year-old patient had onset of fever on October 24, 2003, with asthenia, jaundice, nausea, hematemesis, epistaxis, and gingival hemorrhage. Serologic tests showed IgM against RVFV. No evidence of virus infection was detected in those who had accompanied this patient to the hospital.

In Brakna Province, where 3 cases were confirmed before the investigation (index case-patients 3–5), 6 suspected case-patients (S2–S7) were found during the investigation. Laboratory testing of samples from the 3 index case-patients showed IgM against RVFV by ELISA and RVF viral RNA by RT-PCR. A virus strain (SHM169898) was isolated from index case-patient 4. Among the suspected case-patients from this province, 3 deaths (S2–S4) were recorded, but no samples were available from those patients. Nevertheless, animals in the vicinity of S1, S2, and S3, were found to be infected (5/7, 5/8, and 3/10 animals, respectively). In addition, infection was detected in 4 of 6 persons who had been in contact with suspected case-patient 3. In contrast, no evidence of infection by RVFV was detected in contacts of S2 and S4. The suspected case-patient 5 was a student living in Makhtar Lahjar, who had fever onset on October 20, 2003, was admitted to the National Hospital Center (NHC) of Nouakchott on November 6, and from whose blood RVFV (strain SHM172776) was subsequently isolated. During the investigation, 2 suspected case-patients (S6 and S7) were discovered in the Healthcare Center of Makhtar Lahjar. Suspected case-patient 6 was a 17-year-old female patient who had onset of fever on October 10 and who was admitted to the center on November 1 with headache, abdominal pain, vomiting, hemorrhages, and epistaxis; virus was isolated from this patient (strain SHM172805). The contacts associated with suspected case-patient 6 were also found to be infected with RVFV. Suspected case-patient 7 was a 35-year-old man. He had a fever on October 15 and was admitted to the Health Center of Makhtar Lahjar on November 1 with headache, nausea, vomiting, and epistaxis. The RVF IgM test result for this patient was positive for RVFV.

In Trarza Province, 2 confirmed case-patients were observed during the period of surveillance (index case-patients 6 and 7) and 4 suspected case-patients (S8–S11) were identified during the investigation. Viral isolation was positive for the index case-patient 7 (strain SHM169885). Among the suspected case-patients, 2 (S8 and S9) died before blood samples could be obtained. However, blood testing of samples from 2 animals living in the vicinity of S8 and from 2 human contacts of S9 found recent RVFV infection. Suspected case-patient 10 was 28-year-old man, with onset of fever on October 22, who came for consultation to Keur Macene Healthcare Center. He had a prolonged cough without hemorrhagic symptoms, and an IgM ELISA result for RVFV was positive. The suspected case-patient 11 is a 26 year-old woman, with onset of fever on October 22, who was admitted to the healthcare center of Rkiz with headaches, asthenia and anorexia without hemorrhagic signs. The RVF IgM test result of this patient was positive. In Tagant Province, index case-patient 8, whose condition was diagnosed before the investigation, was a man who came for consultation at the provincial hospital on September 24, exhibiting fever and hematemesis. RVFV (strain SHM169868) was isolated from a blood sample taken on September 29.

In Gorgol Province, 2 suspected case-patients (S12 and S13) were evacuated to the NHC of Nouakchott. The onset of their symptoms dated to October 23 and October 25, respectively. Samples from each were positive for IgM against RVFV by October 30, and RVFV (strain SHM172768) was isolated from S13, who died on October 30.

In Dakar, Senegal, an “imported” case (index case-patient 9) in a person from Rosso, Mauritania, was diagnosed by positive results by ELISA IgM and RT-PCR. This patient was first admitted to the NHC of Nouakchott, Mauritania, before being transferred to the Hôpital Principal de Dakar.

### Animal Cases

Serum samples were obtained and tested from 54 domestic animals (48 goats and 6 sheep) living in the visited localities (Table 1). The median age was 4 years (range 1–15), and the abortion rate was 70% during the last gestation period. IgM against RVFV was detected by ELISA in 25 of 54 animals; no IgG against RVFV was found in any of the 54. Among the animals with a positive test result, the abortion rate was 92%.

**Table 1:** Prevalence rate of immunoglobulin M against Rift Valley fever virus in livestock from Mauritania, 200

District/Locality	Month sampled	Livestock species (no. positive/no. tested)			No. abortions/ no. tested
		Sheep	Goats	Total	
Brakna					
Taiba	Oct	0/1	3/9	3/10	6/10
Guimi	Oct	1/1	4/7	5/8	8/8
Sagle Moure	Nov	0/0	5/7	5/7	5/7
				13/42 (42%)	19/25 (76%)
Trarza					
Boynayé	Oct	0/3	0/6	0/9	0/9
Rkiz	Oct	0/0	2/2	2/2	2/2
				2/11 (18%)	2/11 (18%)
Assaba					
Legrane	Nov	0/1	2/5	2/6	5/6
Kélébélé	Nov	0/0	1/2	1/2	2/2
Tézékré	Nov	0/0	1/2	1/2	2/2
Hseytine	Nov	0/0	6/8	6/8	8/8
				10/18 (55%)	17/18 (94%)
Total		1/6 (16%)	24/48 (50%)	25/54 (39%)	38/54 (70%)

### Mosquitoes

A total of 22,201 mosquitoes, belonging to 4 genera and 17 species, were collected. *Culex poicilipes* was the most frequent species (43.8%), followed by *Cx. antennatus* (23%) and *Mansonia uniformis* (9%). A total of 544 monospecific pools were constituted and submitted for viral isolation. Only *Cx. poicilipes* was found to be associated with RVFV. Three strains (ArD 174367, ArD 174303, and ArD 174347) were isolated from the 146 pools constituted in Guimi Province, giving rise to a minimum infection rate of 0.04% for this locality and 0.01% for the whole study site (Table 2).

**Table 2:** Mosquitoes collected during the Rift Valley fever outbreak, Mauritania, 2003

Locality	*Culex antennatus*		*Cx. poicilipes*		*Cx. tritaeniorhynchus*		*Mansomia uniformis*		Others*		Total	
	C	P	C	P	C	P	C	P	C	P	C	P
K. Macene	273	8	420	12	195	5	1,755	67	365	18	3,008	110
Boynayé	46	1	137	4	766	17	159	5	667	14	1,775	41
Techtayatt	696	14	290	7	451	9	30	1	627	15	2,094	46
Aleg	1,694	35	1437	30	155	3	69	2	435	12	3,790	82
Taiba	2,243	45	255	7	38	2			540	17	3,076	71
Boghe									16	3	16	3
Guimi	159	3	7163	146†					943	21	8,265	170
Legrane			6	1					14	2	20	3
Kélébélé			11	2					23	4	34	6
Hseytine									8	3	8	3
Sarandougu	8	1	18	1	3	1			86	6	115	9
Total	5,119	107	9,737	210	1,608	37	2,013	75	3,724	87	22,201	544

*Aedes. vexans, *Ae. ochraceus*, *Anopheles funestus*, *An. gambiae*, *An. pharoensis*, *An. rufipes*, *An. wellcomei*, *An. ziemanni*, *An. squamosus*, *Cx. ethiopicus*,*Cx. neavei* and *Ma. African*. C, collected; P, pools. †Rift Valley fever virus strains (ArD 174367, ArD 174303, and ArD 174347) isolated in 3 pools.

### Genetic Analysis

**Figure 3:**
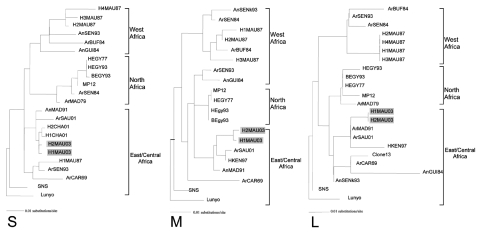
Phylogenetic relationships of the S (small), M (medium), and L (large) RNA segments of Rift Valley fever viruses. Strains isolated in Mauritania (gray shading) are designated H1MAU03 and H2MAU03, according to previous abbreviation guidelines (*24*). Nucleotide sequences of these segments (S, M, and L) have been submitted to GenBank with the following accession nos., respectively: EF160113, EF160116, and EF160117 for H1MAU03; EF160114, EF160115, and EF160118 for H2MAU03. Branch lengths are proportional to the number of substitutions per site.

RNA was extracted from the 8 viral strains isolated from humans, and fragments of the S, M, and L segments were amplified and sequenced. No amino acid (aa) differences were found between the fragments of the S or L segments analyzed (198 and 51 aa, respectively). A single amino acid difference was found between the M fragment (255 aa) of the 5 viral strains analyzed. Results of phylogenetic analyses of the nucleotide sequences of amplified fragments from 3 segments belonging to 2 representative strains (H1MAU03 [SHM169867] and H2MAU03 [SHM169868]) isolated during this epidemic and previously described nucleotide sequences of RVFV are shown in Figure 3. The strains identified in Mauritania 2003 are consistently located within the East/Central lineage for all trees. This lineage contains viral strains that circulated in Madagascar (1991), Kenya (1997), Chad (2001), and Saudi Arabia (2001).

## Discussion

The combination of ELISA, RT-PCR, and isolation assays has permitted the rapid and efficient identification of RVFV as the cause of the extended hemorrhagic fever outbreak reported in Mauritania during the last quarter of 2003. Of the 24 RVF cases diagnosed in the laboratory, 13 were diagnosed by IgM only; 8 were diagnosed by IgM, RT-PCR, isolation, or >1 method; and 3 were diagnosed by RT-PCR, isolation, or both. These data and those obtained during the epidemics of RVF in Kenya (*25*), as well as in Saudi Arabia and Yemen (*4*), demonstrate the importance of combining diagnostic assays for accurate and comprehensive detection of RVFV infection.

Regarding differential diagnosis, only 2 suspected case-patients with fever had confirmed malaria due to infection by *P. falciparum*. This low malaria infection rate suggests that the RVF outbreak was the major cause of the febrile cases notified during this period.

Although WHO estimates that the human mortality rate due to RVFV is ≈1%–2% of infected patients, the number of recorded deaths during this outbreak was 4 among 25 infected patients when the laboratory data were considered exclusively. Epidemiologic investigations have found 5 additional deaths that could be due to RVFV infection. We cannot be absolutely certain about the causes of death in our suspected case-patients from whom no blood sample was taken. However, when the clinical symptoms and the rate of infection in domestic animals are considered, that these cases were the result of RVFV infection is highly probable. In those cases in which the contacts had negative test results and only domestic animals had positive results, we hypothesize that the infection of those with lethal cases was related to socioeconomic/professional activity. Indeed, those at highest risk include butchers and others who come in contact with animals (e.g., slaughterhouse workers, tanners, and herdsmen), who represent a large part of the population living in these areas.

During this investigation, a high infection rate was found in sheep and goats that lived in close proximity to the patients (46.3% of IgM positive compared with 25% during the 1998 outbreak) (*13*). Also, according to interviews with herdsmen, a high abortion rate (92%) was observed in infected animals during their most recent pregnancy. Previous studies showed abortion rates ranging from 80% to 100% (*26*). Nevertheless, the discrepancies observed between the overall abortion rate in animals and the prevalence of anti-RVF IgM (70% vs 46.3%) support the hypothesis of the existence of other cocirculating diseases that also cause abortion. The lack of anti-RVF IgG in domestic animals is surprising considering the long-standing virus endemicity in Mauritania. This observation could be ascribed to different factors: 1) the small number of domestic animals analyzed, 2) a recent introduction of the virus in these localities, or 3) a renewal of animal populations (*27*). The latter hypothesis is supported by the observation of the relatively young median age of the animals tested (4 years old), reflecting a new animal population since the 1998 RVF epidemic in Mauritania.

Among the mosquitoes collected, several species known as RVFV vectors (*Cx. poicilipes, Cx. tritaeniorhynchus, Cx. antennatus*, *An. pharoensis*, *Ae. vexans*, *Ae. ochraceus*, and *Ma. africana*) (*10*,*28*) were recorded, but only *Cx. poicilipes* was found to carry RVFV during the outbreak. Spatial analyses of the results show that *Cx. poicilipes* was in fact predominant only in the village of Guimi, where the RVFV was isolated. This observation indicates that the levels of the different species vary according to the local environment. Mosquitoes from the *Aedes* genus, known for their role in RVFV maintenance and transmission, were scarce, likely due to their early appearance at the onset of the rainy season, whereas our investigation took place at the end of the rainy season. Indeed, in 2003, the last rainfall event was recorded at the beginning of October.

Genetic analyses of the 3 segments of RVFV isolated during this epidemic showed a low level of variation between isolates from the different provinces. This finding supports the hypothesis that the same strain was circulating in the different affected areas. The nucleotide sequences of the strains isolated during this epidemic compared with those isolated elsewhere in Africa and Saudi Arabia showed that they belong to the East/Central African cluster for the 3 segments. Previous reports have shown that some strains isolated in West Africa share 1 or 2 segments with strains belonging to the East/Central African cluster (*2**,**29*). However, to our knowledge, this is the first evidence of the circulation in West Africa of strains harboring 3 segments that all belong to the East/Central African cluster.

This finding confirms the existence of RVFV strain exchanges between geographic areas. In fact, the spread of RVFV from East Africa to other regions has already been observed during the RVF outbreak in Saudi Arabia and Yemen in 2000–2001 (*4*) and in Chad in 2001 (*30*). RVFV was also found to be the cause of the epidemic/epizootic in Egypt in 1977 and in Madagascar in 1979 (*31*). Such a mechanism of RVFV spread likely depends on human and animal population movements for which animal migration routes between West and East/Central Africa need to be identified. Furthermore, previous studies have demonstrated that reassortant viruses can emerge when 2 RVFV lineages coexist (*24*). Favorable environmental conditions (mainly the rainfall pattern), which led to the emergence of already introduced East/Central African strains, seem to be the cause of this outbreak. Indeed, RVFV emergence in East Africa is undoubtedly the consequence of rainfall surplus (*32*,*33*). In contrast, in West Africa, the few studies carried out in the past indicate that rainfall surplus is not a key factor for RVFV emergence. In fact, RVF outbreaks were often observed during years of rainfall deficit (*10*,*34*). Therefore, this outbreak in Mauritania, caused by RVFV strains of the East/Central African lineage, is likely linked to the heavy rainfall recorded during 2003 in the affected areas (315 mm in 2003 versus 161 mm in 2002). These arguments support the hypothesis that episodes of heavy rainfalls are directly or indirectly more favorable to the emergence of virus strains belonging to the East/Central African cluster. However, such a hypothesis presumes the existence of ecologic or biologic differences between strains of the 2 lineages, and further investigations are needed with special emphasis on the interactions with the strains’ respective vectors and reservoirs.
